# Reprogramming of fibroblasts to uterine glandular epithelium by a chemical cocktail induction

**DOI:** 10.1038/s41421-019-0096-8

**Published:** 2019-05-14

**Authors:** Xuewei Yuan, Zhengquan He, Junjie Mao, Yanping Hu, Xin Liu, Jia Guo, Zhonghua Liu, Ying Zhang, Wei Li, Qi Zhou

**Affiliations:** 1 College of Life Science, Northeast Agricultural University, Harbin, 150030 China; 2 State Key Laboratory of Stem Cell and Reproductive Biology, Institute of Zoology, Chinese Academy of Sciences, Beijing, 100101 China; 3 Institute for Stem Cell and Regeneration, Chinese Academy of Sciences, Beijing, 100101 China; 4 University of Chinese Academy of Sciences, Beijing, 100049 China

**Keywords:** Transdifferentiation, Transdifferentiation

Dear Editor,

Absolute uterine factor infertility (AUFI) affects 3–5% women and has multifactorial causes. Uterus transplantation is the main treatment option for AUFI^1^. Alternatively, artificial uterus can be used as an incubator for embryos. A bioartificial uterus requires a complete structure that includes cell components; however, poor cell sources hamper the clinical applications of bioartificial uterus.

Mammalian uterus glandular epithelium (GE) is necessary for blastocyst implantation and its deficiency can lead to infertility^2^. GE responds to the ovarian hormones, estrogen (E2), and progesterone (P4), and induces the expression of leukemia inhibitory factor (Lif) for the embryo implantation^3,4^. Direct reprogramming of the fibroblasts by specific transcription factors or chemical molecules have been previously reported^5,6^. However, obtaining uterine cells through fibroblasts reprogramming has rarely been reported. In this study, we reported the generation and characterization of full chemical-induced uterine GE from fibroblasts with the ability of amplification in vitro and responding to ovarian hormones. Our study identified a new cell source that can be used for the reconstruction of the uterus or other organs and suggested a potential value for treating uterine infertility and the related reproductive medical disorders.

Mouse embryonic fibroblasts (MEFs) were isolated from embryos at embryonic day 13.5 (E13.5) and then cultured with a chemical cocktail consisting of FGF2, BMP4, mLif, 1,4-DPCA (a competitive inhibitor of HIF prolyl 4-hydroxylase), A8-301 (an inhibitor of TGF-β type I receptor ALK5 kinase), and CHIR-99021 (a GSK-3α/β inhibitor)-inducing medium (FBLDAC). Briefly, MEFs were cultured in the inducing medium for 12 days, then maintained in the expanding medium (see Supplementary methods) (Fig. 1a). Finally, the FBLDAC-induced epithelial cell-like colonies were obtained (Fig. 1b). Furthermore, these colonies could be passaged for more than 20 times with a uniform colony morphology on the matrigel- or 10% FBS-coated petri dish (Fig. 1c) and a stable karyotype of “38 + XX” or “38 + XY” (Fig. 1d, Supplementary Fig. S1a). These cells expressed the specific markers of epithelial cells including KRT19, EPCAM, CDH1 and proliferative protein, KI67 (Fig. 1e). Next, we evaluated the tumorigenicity by subcutaneous transplantation of the induced cells and mouse embryonic stem cells (ESCs) in nude mice. Our results showed that there was no teratoma formation among the ten mice that were transplanted with the induced cells for 2 months, while eight teratomas formation was observed in the ten mice transplanted with mouse ESCs for 3 weeks (Fig. 1f).

**Fig. 1:**
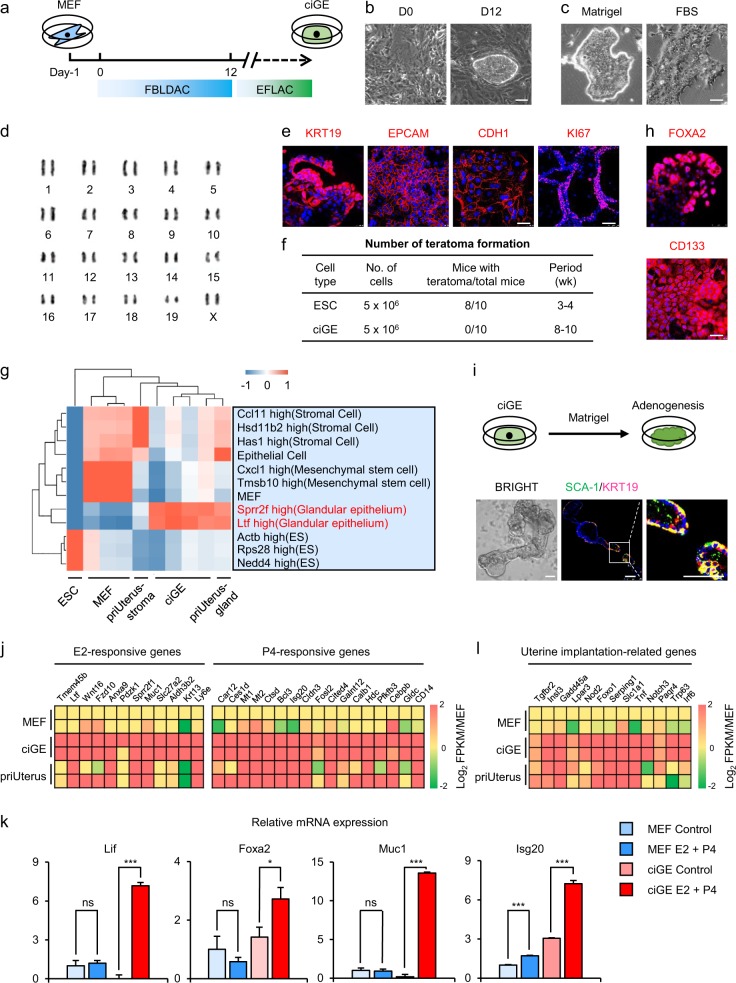
The reprogramming of MEFs into ciGEs by a chemical cocktail. **a** The scheme of reprogramming of MEFs into ciGEs by a chemical cocktail. MEFs were plated on 0.1% gelatin in fibroblast growth medium for 1 day, then the medium was changed into inducing medium containing FBLDAC chemical cocktail. At day 12, the medium was changed into expanding medium containing EFLAC chemical cocktail. The first colony appeared on the induced medium during day 8 to day 12. **b** Representative morphologies of MEFs and MEF-derived epithelial colonies induced by the FBLDAC chemical cocktail. Scale bar, 100 µm. **c** Representative morphologies of MEF-derived epithelial colonies on matrigel (left)- and 10% FBS (right)- coated dish. Scale bar, 75 µm. **d** Karyotype analysis of MEF-derived epithelial cells with “38 + XX” karyotype after more than 20 generations. **e** Immunostaining of pan epithelial cell markers KRT19, EPCAM, and CDH1, scale bar, 50 µm, and cell proliferation marker KI67 in MEF-derived epithelial cells. Scale bar, 100 µm. **f** Ratio of tumor formation in nude mice. **g** Heatmap and hierarchical clustering of genes from RNA-Seq data. Samples of MEFs, ciGEs, and priUterus (separated from adult uterine) were used to compare with cell types in MCNA. **h** Immunostaining of FOXA2 (upper panel) and CD133 (lower panel) in ciGEs. Scale bar, 50 µm. **i** Bright-field images and immunostainings show the ability of self-assembly into a cavity structure of ciGEs. Scale bar, 75 µm. **j** E2- and P4-responsive genes were significantly upregulated in ciGEs, compared to MEFs. See also Supplementary Fig. S3d. **k** Relative mRNA expression of E2- and P4-responsive genes in MEFs and ciGEs after E2 and P4 treatment. *p < 0.05; **p < 0.01; ***p < 0.001; *p* > 0.05 were considered nonsignificant, Student’s *t*-test. Data are presented as the mean ± S.D., *n* = 3. **l** Genes involved in uterine implantation were upregulated in ciGEs, compared to MEFs

To confirm that the epithelial cells were indeed induced from MEFs, we isolated MEFs from transgenic mice carrying fibroblast-specific protein 1 (Fsp1)-Cre/ROSA26^mTmG^ to trace the fibroblasts. The fibroblasts would permanently express the membrane-targeted GFP after the Fsp1-Cre-mediated excision of membrane-targeted tomato expression (Supplementary Fig. S1b). These traced cells ruled out contamination of cells with epithelial fate (Supplementary Fig. S1c) and were induced according to the process shown in Fig. 1a. The green colonies of the induced cells emerged after the induction by FBLDAC (Supplementary Fig. S1d). The epithelial fate of the induced cells was confirmed by immunofluorescence (Supplementary Fig. S1e).

To further characterize these induced epithelial cells, we sequenced the whole transcriptome of the chemical-induced glandular epithelial cells (named as ciGEs hereinafter), MEFs and primary epithelial cells isolated from mouse uterus (priUterus). Hierarchical clustering analysis revealed that the gene expression patterns of ciGEs were closely related to priUterus, while differed significantly from MEFs (Fig. 1g). Furthermore, ciGEs have high expression levels of *Sprr2f* and *Ltf*, similar to GE^7^ (Fig. 1g). With Gene Ontology (GO) analysis, we found that genes expressed in ciGEs were related to the uterus development and estrogen response. We found that ciGEs were mainly enriched with GO terms associated with epithelial fate and functions when compared with MEFs (Supplementary Fig. S2) which is indicative of the uterine glandular epithelial characteristics of ciGEs. We further detected the high expression of uterine glandular epithelial specific markers (FOXA2 and SOX17) (Fig. 1h, upper panel, Supplementary Fig. S3a) and adult stem/progenitor cell markers (Fig. 1h, lower panel, Supplementary Fig. S3b) in ciGEs. Moreover, when partially digested, ciGEs formed a gland-like structure with lumen (Fig. 1i), which implies that the ciGEs can undergo adenogenesis. We also detected the upregulation of E2- and P4-responsive genes in ciGEs (Fig. 1j, Supplementary Fig. S3c and d), which suggested that the ciGEs can respond to ovarian hormones. Additionally, hormone treatment influenced the expression dynamics of E2- and P4-responsive genes, including Lif (Fig. 1k), a critical regulator expressed in the uterine glands to promote embryo implantation^8^. Besides, we also found that the upregulation of implantation-related genes (Fig. 1l). These results suggested an expandable and functional uterine GE fate can be induced from fibroblasts by a chemical cocktail.

In all mammals, the uterus contains glands in the endometrium and becomes receptive when the embryo is ready to be implanted. At this stage, the uterine environment is conducive to the embryo growth and is mainly regulated by E2 and P4^9^. Uterine glands and their secretions are necessary to establish uterine receptivity and embryo implantation, early pregnancy failure is usually caused by the secretory phase defect^10^. In this study, we obtained ciGEs with uterine glandular epithelial characteristics and which respond to ovarian hormones, implying their application in endometrium replacement in clinical treatment. CiGEs were obtained by induction of fibroblasts using only a chemical cocktail. The chemical cocktail has several advantages including cell permeability, convenient handling, being nonimmunogenic, and ease of standardization. These advantages make it an attractive strategy for clinical application in treatment of uterine diseases like AUFI. Furthermore, we found the upregulation of functional related genes including estrogen and progesterone response genes in ciGEs. The molecular mechanism of the chemical cocktail-induced trans-differentiation needs to be explored in the future. Nevertheless, our research provides a clue to generate target cells from resident fibroblasts in situ in damaged or aging uterus through an induction by a chemical cocktail. It also provides an in vitro model for the study of embryo implantation and loss of uterine structure or function. Meanwhile, our results provide new insights into treatments for uterine factor infertility and uterine regeneration.

## Supplementary information

Supplementary information

## Supplementary information

**Supplementary information** accompanies the paper at (10.1038/s41421-019-0096-8).
