# Supplementary material for: Reprogramming of fibroblasts to uterine glandular epithelium by a chemical cocktail induction
Source: Cell Discov. 2019 May 14;5:26. doi: 10.1038/s41421-019-0096-8 (PMC6514004; doi:10.1038/s41421-019-0096-8)
Supplement: Supplementary file 1 — Supplementary information [file 41421_2019_96_MOESM1_ESM.pdf]

Supplementary Fig. S1

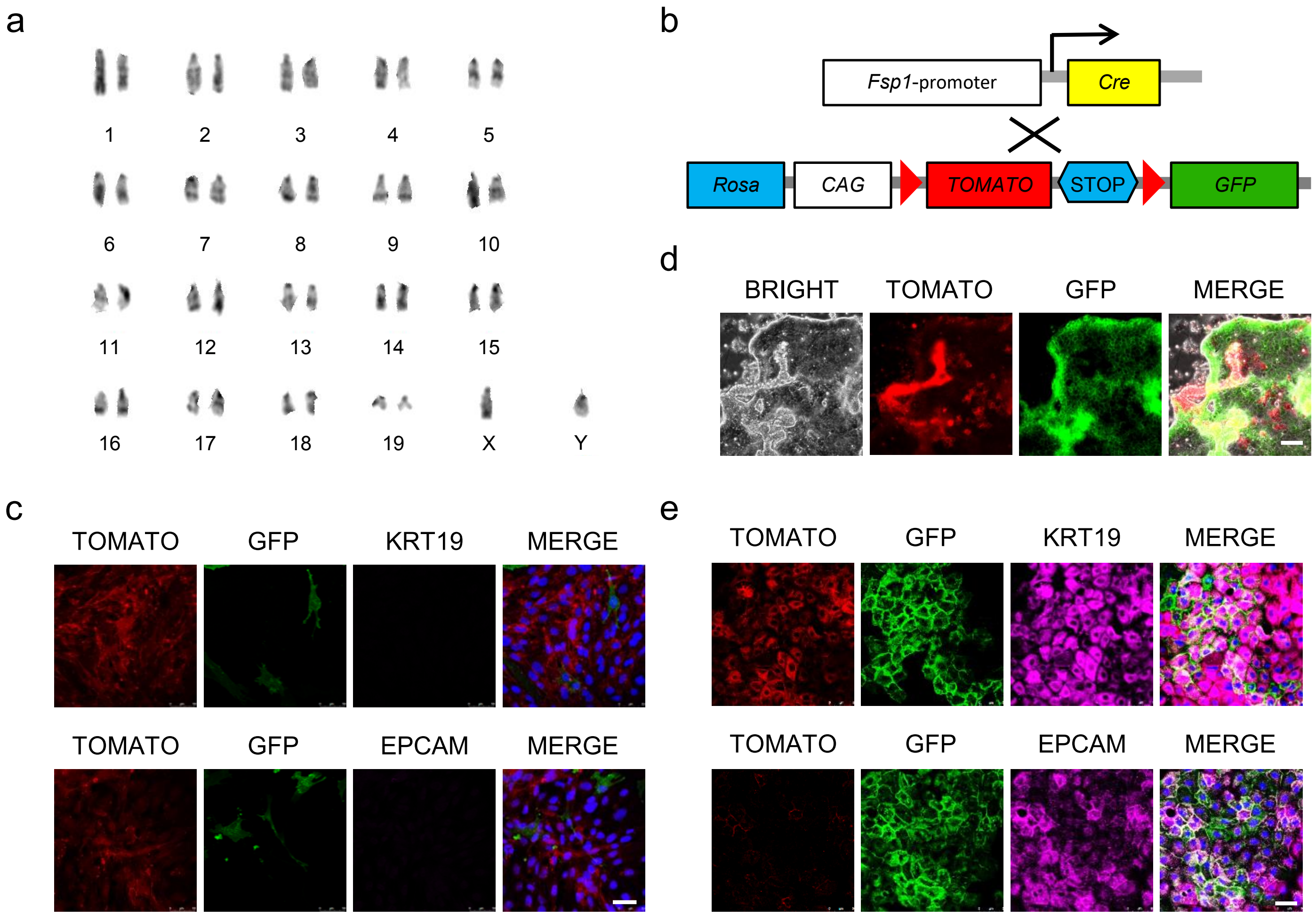

Supplementary Fig. S2

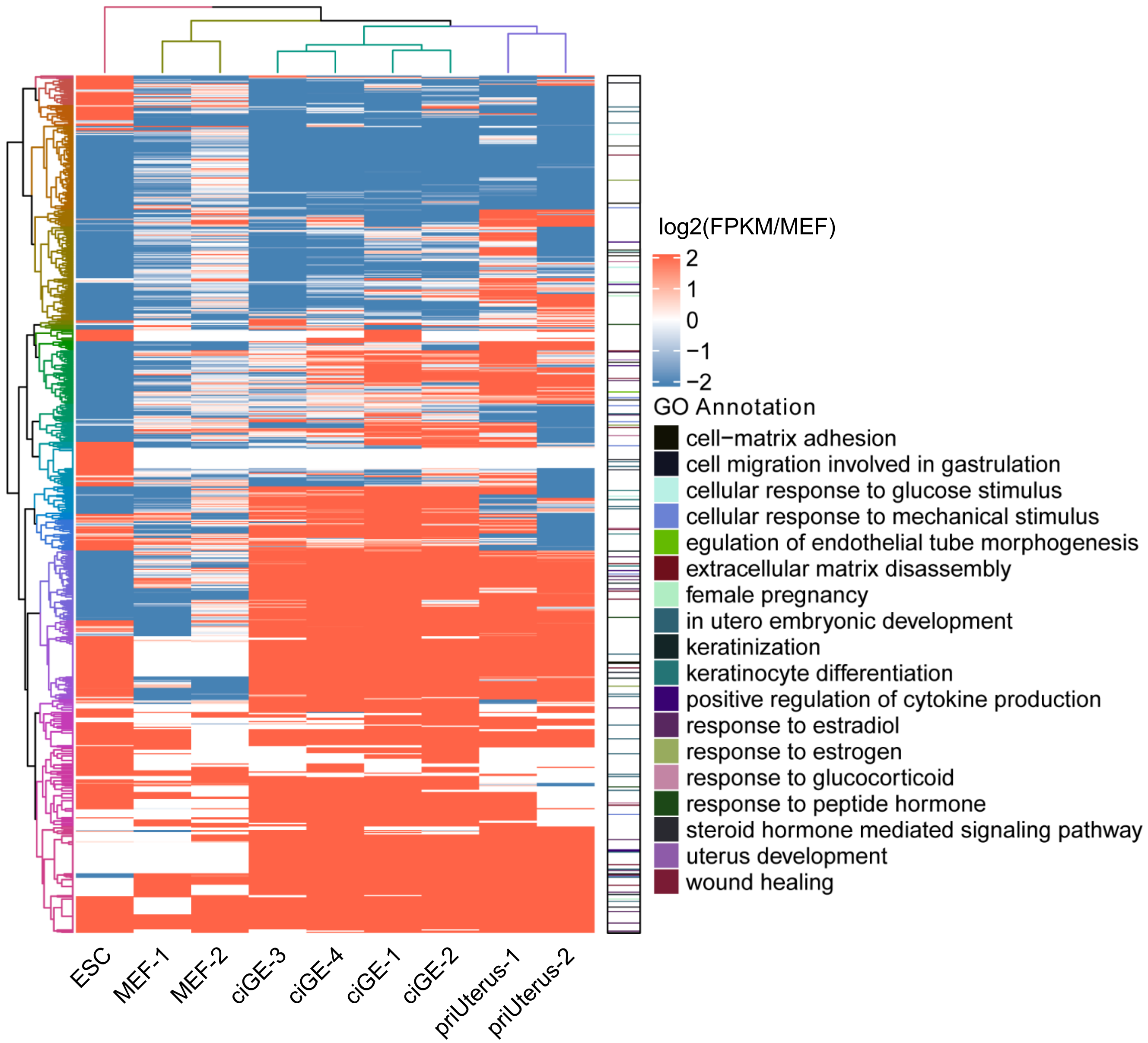

Supplementary Fig. S3

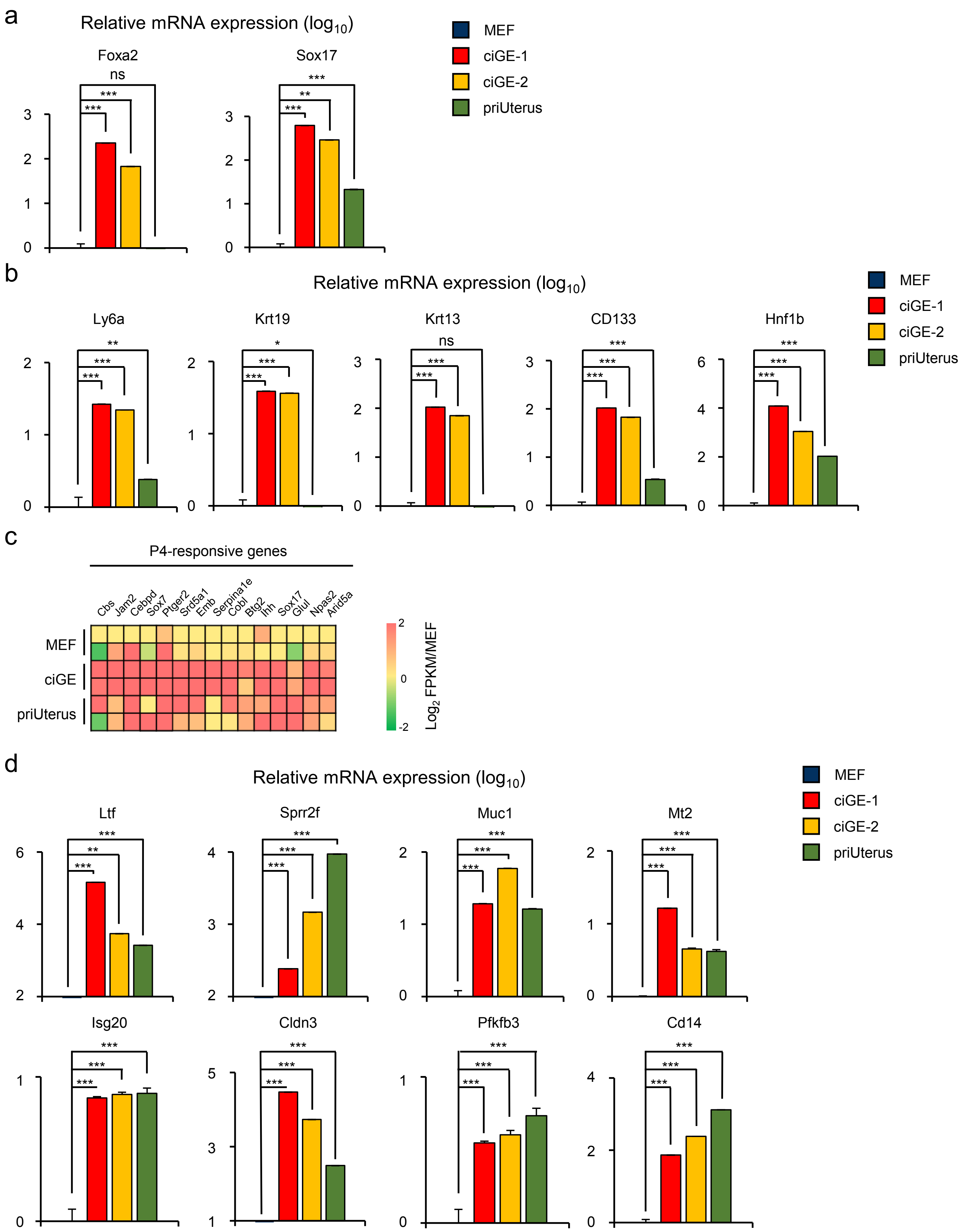

Supplemental Fig. S1 Direct reprogramming of MEFs into ciGEs by a chemical cocktail. **a** Karyotype analysis of MEF-derived epithelial cells with "38 + XY" karyotype after more than 20 generations. **b** Schematic of obtaining Fsp1-Cre/R26R^{mTmG} MEFs. **c** Induction of epithelial colonies from Fsp1-Cre/R26R^{mTmG} MEFs. Scale bar, 50 μm. **d** Immunostaining of pan epithelial cell markers, KRT19 and EPCAM, in Fsp1-Cre/R26R^{mTmG} MEFs. Scale bar, 50 μm. **e** Immunostaining of pan epithelial cell markers, KRT19 and EPCAM, in Fsp1-Cre/R26R^{mTmG} MEFs derived epithelial cells. Scale bar, 50 μm.

Supplemental Fig. S2 Expression heatmap and Gene ontology analysis of differentially expressed genes (DEGs). FPKM values of DEGs were normalized to MEFs, and each line in the rightmost box represented a GO term of the gene in the heatmap.

Supplemental Fig. S3 Characteristics of the glandular epithelium fate. **a, b and d** Relative mRNA expression of genes involved in the uterine epithelial, adult stem/progenitor cells, and hormone response. *p < 0.05; **p < 0.01; ***p < 0.001; p > 0.05 were considered non-significant (ns), Student's *t* test. Data are presented as the mean ± S.D., *n* = 3. **c** P4-responsive genes were significantly up-regulated in ciGEs, compared to MEFs. See also Fig. 1j.

Materials and Methods

Animal Experiments. Specific-pathogen-free (SPF)-grade mice were obtained from Vital River Laboratories, Beijing, and were housed in the animal facilities of the Chinese Academy of Sciences, Bejing, China. All the studies were carried out in accordance with the Guidelines for the Use of Animals in Research issued by the Institute of Zoology, Chinese Academy of Sciences. Five-week-old BALB/c Nude male mice were used in tumorigenic experiment.

Eight-week-old C57BL/6J mice were used for embryo collection. Fsp1-Cre transgenic mice (012641) and ROSA$^{mT/mG}$ mice (007676) which have C57BL/6J congenic background were purchased from Jackson Laboratories (USA). ROSA$^{mT/mG}$ is a cell membrane-targeted, two-color fluorescent Cre-reporter allele. Without Cre recombination, membrane-localized tdTomato (mT) fluorescence expression is widespread in cells/tissues, and Cre recombinase expressing cells (and future cell lineages derived from these cells) express membrane-localized EGFP (mG) fluorescence replacing the red fluorescence.

Cell induction and culture in the vitro. Mouse embryonic fibroblasts (MEFs) were isolated from fetal mice of embryo 13.5 days (E13.5). The cultured fibroblasts were treated in a chemical cocktail, FBLDAC [F, FGF2 (R&D, 233-FB-001MG/CF, 20 ng/mL); B, BMP4 (R&D, 233-FB-001MG/CF, 10 ng/mL); L, mLif (Millipore, ESG1007, 1000 U/mL); D, 1,4-DPCA (Enzo, BML-EI377-0050, 5 μM); A, A 83-01 (Stemgent, 04-0014, 10 μM); C, CHIR99021 (Stemgent, 04-0004, 12 μM)) inducing medium. Briefly, fibroblasts were cultured in DMEM medium (Gibco, C11995500BT) with 10% FBS (Gibco, 16000-044) for 24 hours. The cultured fibroblasts were then transferred into an inducing medium that contains the FBLDAC cocktail and other supplements including DMEM/F12 (Gibco, 12400-024) and Neurabasal (Gibco, 21103-049) (1:1 mixture), N2 (Gibco, 17502-048), B27 (Gibco, 17504-044), 10% FBS and 10% KOSR (Gibco, 10828028)]. The inducing medium was changed every 3 days. After inducing for 12 days, cells were maintained on matrigel (BD, 354277) or 10% FBS coated dish in the expanding medium [DMEM/F12 and Neurabasal (3:1), 2% KOSR 100 ng/mL EGF (R&D, 2028-EG-200), 10 ng/mL FGF2, 1000 U/mLif, 5 μM A 83-01, 3 μM CHIR99021, 1 μg/mL Heparin (Sigma, H4784), 55 μM

β-mercaptoethanol (Gibco, 21985), 0.002% BSA (Sigma, A7906-100G), NEAA (Gibco, 11140-050, 100 ×), N2 and B27]. Trypsin (0.25%) (Gibco, 25200072) digestion was done to passage the cells, the passage ratio was 1: 4 to 1: 6.

Karyotype analysis. Karyotype analysis was performed as previously reported [1]. Briefly, ciGEs were incubated with 0.2 mg/mL nocodazole (Sigma, M1404) for 4 hours. The ciGEs were suspended in 0.075 M KCl at 37°C for 30 min after trypsinization. Cells were fixed with a solution consisting of methanol and acetic acid (3:1 in volume) for 30 min and then were dropped onto the pre-cleaned slides. The cells were stained with Giemsa stain (Sigma, GS500ML) for 15 min after being incubated in 5 M HCl.

Detection of tumorigenicity. Approximately 5×10^6 ESCs and ciGEs were injected subcutaneously into the hind limbs of 5-week-old BALB/c Nude male mice, respectively. Teratoma or tumors were monitored 3 weeks later.

RNA-Seq data analysis. To assess the transcriptome of each sample, *Tuxedo Suite* was employed during the analysis of RNA-Seq data. Briefly, cleaned row sequencing reads were aligned to GRCm38 from ensemble release 92 by *Tophat* (*Bowtie2*) with default parameters, and then mapped reads were used to assemble the transcripts and calculate FPKM values using *Cufflinks*. For differentially expressed genes (DEGs) analysis, *Cuffdiff* with appropriate parameters were adopted, and DEGs with significant threshold 0.05 were selected for the further cell type clustering. We used tool scMCA to match cell types of each sample in the data base MCNA [2], and functional annotation and enrichment of DEGs were performed by David [3]. The raw sequence data reported in this paper have been deposited in the Genome Sequence Archive [4] in BIG Data Center [5], Beijing Institute of Genomics (BIG), Chinese

Academy of Sciences, under accession number CRA001472 that is publicly accessible at http://bigd.big.ac.cn/gsa.

Immunofluorescence. Cells were cultured on glass coverslips and then fixed with 4% PFA for 1 hour, subsequently washed with PBS for 3 times. The cells were blocked with 0.1% Triton X-100 for 30 min and 2% BSA for 1 hour at room temperature (RT). The cells were then incubated with the primary antibodies at 4°C overnight and then with species-specific secondary antibodies for 1 hour at RT. The cells were incubated with DAPI for 10 min at RT. The images were taken by the confocal microscope (Leica TCS Sp8). The primary antibody information used is as follows: anti-KRT19 (Rabbit, Abcam, Ab52625, 1:500), anti-EPCAM (Rabbit, Abcam, Ab71916, 1:500), anti-CDH1 (Rat, Sigma, U3254, 1:500), anti-KI67 (Rabbit, Thermo Fisher Scientific , PA5-19462, 1:200), anti-FOXA2 (Goat, SantaCruz, Sc-9187, 1:200), anti-CD133 (Rabbit, Abcam, Ab16518, 1:500), anti-SCA-1 (Rat, Abcam, Ab51317, 1:500), anti-CDX2 (Rabbit, Cell Signaling Technology, 3977s, 1:200).

Quantitative RT-PCR. Cell total RNA was extracted with TRIzol reagent (Invitrogen, 15596-018). High Capacity cDNA Reverse Transcription Kit (ABI, 4368814) was used to reverse transcription of cDNA. Relative gene expression was analyzed based on the 2-ΔΔCt method with GAPDH as internal control. All primers are listed as following:

Gapdh forward primer	5′-AGGTCGGTGTGAACGGATTTG-3′
Gapdh reverse primer	5′-TGTAGACCATGTAGTTGAGGTCA-3′
Sprr2f1 forward primer	5′-GTCTTCAGGACAGGGGAAAGA-3′
Sprr2f1 reverse primer	5′-CCCCTTTACAGCAGCAAGAT-3′

Ltf forward primer	5′-TGAGGCCCTTGGACTCTGT-3′
Ltf reverse primer	5′-ACCCACTTTTCTCATCTCGTTC-3′
Muc1 forward primer	5′-GGCATTCGGGCTCCTTTCTT-3′
Muc1 reverse primer	5′-TGGAGTGGTAGTCGATGCTAAG-3′
Foxa2 forward primer	5′-CCCTACGCCAACATGAACTCG-3′
Foxa2 reverse primer	5′-GTTCTGCCGGTAGAAAGGGA-3′
Lif forward primer	5′-ATTGTGCCCTTACTGCTGCTG-3′
Lif reverse primer	5′-GCCAGTTGATTCTTGATCTGGT-3′
Isg20 forward primer	5′-TCTTGGGGGTTGGAGGATG-3′
Isg20 reverse primer	5′-CGGAGGTAGAAAGGGCGTC-3′
Sox17 forward primer	5′-GATGCGGGATACGCCAGTG-3′
Sox17 reverse primer	5′-CCACCACCTCGCCTTTCAC-3′
Ly6a forward primer	5′-GAGGCAGCAGTTATTGTGGAT-3′
Ly6a reverse primer	5′-CGTTGACCTTAGTACCCAGGA-3′
Krt19 forward primer	5′-GGGGGTTCAGTACGCATTGG-3′
Krt19 reverse primer	5′-GAGGACGAGGTCACGAAGC-3′
Krt13 forward primer	5′-GCCAGCTACCTGGATAAGGTG-3′
Krt13 reverse primer	5′-CAGATGCCAGTCACGAATCTTC-3′
CD133 forward primer	5′-CCTTGTGGTTCTTACGTTTGTTG-3′
CD133 reverse primer	5′-CGTTGACGACATTCTCAAGCTG-3′
Hnf1b forward primer	5′-CCCCTCACCATCAGCCAAG-3′
Hnf1b reverse primer	5′-GGTTCTGAGATTGCTGGGGATT-3′

Mt2 forward primer	5′-GCCTGCAAATGCAAACAATGC-3′
Mt2 reverse primer	5′-AGCTGCACTTGTCGGAAGC-3′
Cldn3 forward primer	5′-ACCAACTGCGTACAAGACGAG-3′
Cldn3 reverse primer	5′-CAGAGCCGCCAACAGGAAA-3′
Pfkfb3 forward primer	5′-CCCAGAGCCGGGTACAGAA-3′
Pfkfb3 reverse primer	5′-GGGGAGTTGGTCAGCTTCG-3′
Cd14 forward primer	5′-CTCTGTCCTTAAAGCGGCTTAC-3′
Cd14 reverse primer	5′-GTTGCGGAGGTTCAAGATGTT-3′

Self-assembling of glands. CiGEs were partly digested with 0.25% trypsin for 1 min to avoid producing single cells. The cell aggregates were gently aspirated with a pipette and inoculated on a petri dish pre-coated with matrigel and cultured in the expanding medium for 7-14 days.

Hormone response experiments. For estrogen and progesterone stimulation, ciGEs or MEFs were cultured with expanding medium with mLif, N2 and B27 removed. 8 nM β-estrogen (Sigma, E8875) and 200 ng/mL progesterone (Sigma, p8811) or DMSO (Sigma, D2650) were administrated for 3 days, and the mRNA was collected to identify gene expression.

Statistical analysis. Statistical analysis was performed using Prism Software (GraphPad). Data shown are presented as the mean ± SD of three or more independent experiments. A Student's *t* test was used for statistical analysis of data.

References

1. He, Z.-Q. *et al.* Generation of Mouse Haploid Somatic Cells by Small Molecules for Genome-wide Genetic Screening. *Cell Rep* **20**, 2227-2237 (2017).
2. Han, X. *et al.* Mapping the Mouse Cell Atlas by Microwell-Seq. *Cell* **173**, 1307 (2018).
3. Huang da, W., Sherman, B. T. & Lempicki, R. A. Systematic and integrative analysis of large gene lists using DAVID bioinformatics resources. *Nature Protoc* **4**, 44-57

(2009).
4. Wang, Y. *et al.* GSA: Genome Sequence Archive*. *Genomics, Proteomics & Bioinformatics* **15**, 14-18 (2017).
5. BIG Data Center Members. Database Resources of the BIG Data Center in 2018. *Nucleic Acids Res* **46**, 14-20 (2017).
